# Giant lumbar schwannoma with retroperitoneal extension and vertebral body invasion: a case report

**DOI:** 10.11604/pamj.2023.44.140.37717

**Published:** 2023-03-17

**Authors:** Angky Saputra, Joshua Sutikno, Raka Janitra, Erliano Sufarnap, Centery, Roslan Yusni Hasan, Satyanegara, Harviandi Sumarsudi, Franky Yesaya Siahaan, Komaruddin Boenjamin

**Affiliations:** 1 Neurosurgery Department, Mayapada Hospital, Tangerang, Indonesia,; 2 General Surgery Department, Mayapada Hospital, Tangerang, Indonesia,; 3 Thoracic and Cardiovascular Surgery Department, Mayapada Hospital, Tangerang, Indonesia,; 4 Urology Department, Mayapada Hospital, Tangerang, Indonesia

**Keywords:** Schwannoma, lumbar spine, retroperitoneal, surgery, case report

## Abstract

Schwannoma is a type of peripheral nerve sheath tumor derived from Schwann cells. There have been only a few cases of giant lumbar schwannoma with retroperitoneal extension eroding the vertebral body documented. Thus, managing these tumors presents various challenges. This paper reports a case of a 59-year-old woman who experienced lower back radicular pain for a year. A lumbar magnetic resonance imaging revealed the presence of a giant extradural soft tissue tumor measuring 8.6x7.4x9.7 cm, compressing the right L5-S1 neural foramen and extending into the retroperitoneal space while eroding the L5 vertebral body. The patient underwent surgery via a retroperitoneal approach, and the tumor was successfully resected. Histopathological examination confirmed the diagnosis of schwannoma. In conclusion, giant retroperitoneal lumbar schwannomas with bone invasion are rare, and gross total resection is the preferred treatment option, but the size and location of the tumor can make the surgery challenging.

## Introduction

Schwannoma is a peripheral nerve sheath tumor derived from Schwann cells. It is a slow-growing benign tumor, often well-encapsulated and seldom invasive [1]. Furthermore, its most common location is the intracranial region, followed by the spine [1]. Spinal schwannomas are estimated at 0.3-0.4/100,000 per year [1], of which most are intradural-extramedullary, with some being extradural [1]. Those in the retroperitoneal space are rare, accounting for only 3-5% of all schwannomas [2].

The giant types with bony invasion are also rare due to their slow growth and benign nature [2]. Only a few cases of giant lumbar schwannomas with retroperitoneal extension and vertebral body erosion have been published in English literature [2]. This report presents the case of a 59-year-old woman with a giant lumbar schwannoma with retroperitoneal extension and vertebral body invasion. This case is unique due to its size, rarity, and the complexities involved in its management.

## Patient and observation

**Patient information:** a 59-year-old woman presented with chronic radicular pain in her lower back that radiated to her right leg, including pain, numbness, and tingling sensations in the lateral part of her right thigh, calf, and foot. Despite treatment with nonsteroidal anti-inflammatory drugs and pregabalin, her symptoms persisted for approximately one year. The patient had no history of urinary or bowel disturbance or prior trauma, and her medical and family history were unremarkable.

**Clinical findings:** the patient reported a pain intensity of 7 out of 10 on the Visual Analogue Scale (VAS). A physical examination revealed paraesthesia in the right L5 dermatome and positive results on the straight leg raise test at a 45-degree angle in her right foot. Furthermore, no pathological reflexes, abnormalities in muscle tone, muscle weakness, or foot drop were observed during the examination.

**Figure 1:**
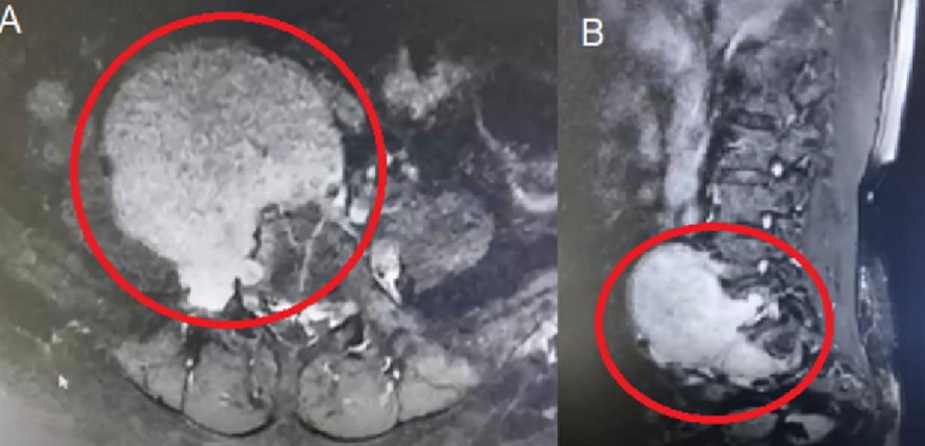
A) a heterogenous contrast-enhancing mass (indicated within red circle) from the L5 nerve root extending to retroperitoneal space and eroding some of the L5 vertebral body; B) the mass (indicated within red circle) originated from L5 nerve root extending to retroperitoneal space with craniocaudal length >2 vertebral bodies

**Diagnostic assessment:** a lumbar Magnetic Resonance Imaging (MRI) revealed a giant mass at the paravertebral L5-S1 vertebral bodies in a non-contrast image. The MRI with contrast showed the presence of an extradural soft tissue tumor, measuring 8.6x7.4x9.7 cm, compressing the L5-S1 intervertebral foramen and extending into the retroperitoneal space, as well as compressing the right iliac artery, as shown in Figure 1. The tumor had an oval shape, well-defined borders and showed heterogeneous contrast enhancement. It eroded the L5 vertebral body and transverse process. Furthermore, no spondylolisthesis or malalignment was observed in the vertebral column. The tumor's massive growth and bony invasion led to the suspicion that it was a chordoma. The result of all blood laboratory data was within normal range.

**Figure 2:**
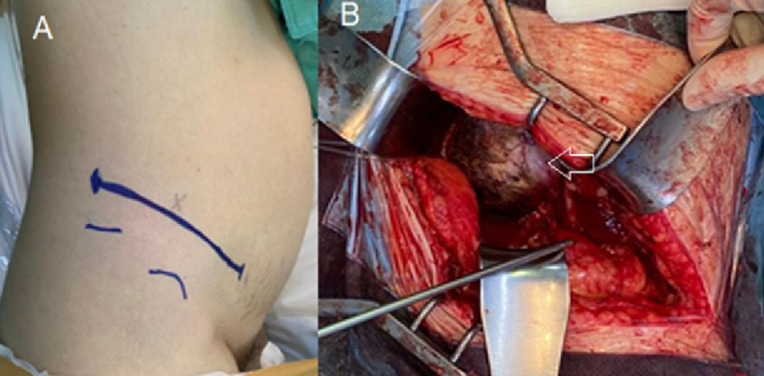
A) preoperative photo showing incision marking and preparation for retroperitoneal approach; B) intraoperative photo showing a white-greyish mass with well-defined edges was found in retroperitoneal space (indicated by a white arrow)

**Therapeutic intervention:** a multidisciplinary team, comprising a neurosurgeon, general surgeon, urologist, and vascular surgeon, was assembled to plan and execute the surgical removal of the giant tumor from the patient. The vascular surgeon was stationed in the operating room to address any potential vascular complications that may arise during the procedure. Before the surgery, the ureteric stent was placed to avoid ureteral injury during resection. The retroperitoneal approach of the lumbar spine was used in the left lateral decubitus position. The L5 vertebral body and disc space were identified with C-arm fluoroscopy and then marked on the patient's skin, as shown in Figure 2A.

Furthermore, intraoperative neurophysiological monitoring using electromyography was applied to confirm neurologic changes perioperatively. A longitudinal incision was made 2 inches anterior from the L2 disc space level. This was carried down to the subcutaneous level, and muscle dissection was performed to identify the peritoneum. The external oblique, internal oblique, and transverse abdominal muscles were identified and split along the muscle lining. After identifying the peritoneum, blunt dissection was performed down to the retroperitoneal space using a sponge stick, as shown in Figure 2B. The tumor was identified, and a surgical microscope was used to visualize the tumor which was located anterior to the psoas muscle. It was well-defined, with greyish-white color, solid, and hard to move. Once the safety margins were defined using a stimulator probe, the capsule was incised and removed using the piecemeal method. Meticulous bleeding control and careful retraction were also conducted during the debulking when the right common iliac vein was injured and repaired by the vascular surgeon. The base of the tumor was discovered at the L5-S1 intervertebral foramen and was suspected to be originating from the nerve sheath of the L5 nerve root.

Additionally, the tumor also infiltrated part of the L5 vertebral body, and it was excised. It was confirmed that no residual was left at the surgery site. Electrocauterization was performed to control bleeding, and suturing was performed by anatomical layer. Finally, the total blood loss during the surgery was 500 cc.

**Follow-up and outcomes:** following the surgical procedure, the patient was admitted to the Intensive Care Unit (ICU). A blood transfusion, consisting of one bag of packed red cells, was administered due to blood loss during surgery and a hemoglobin level of 7 g/dL. The patient was monitored for 24 hours before being transferred to the surgical ward upon removal of the ureteric stent on the second postoperative day. There was no evidence of venous thrombosis, neurological deficit, or other complications.

A consultation was conducted with a medical rehabilitation specialist for physiotherapy and exercise to aid in the patient's mobilization. Finally, the patient was discharged a week after the surgery.

**Figure 3:**
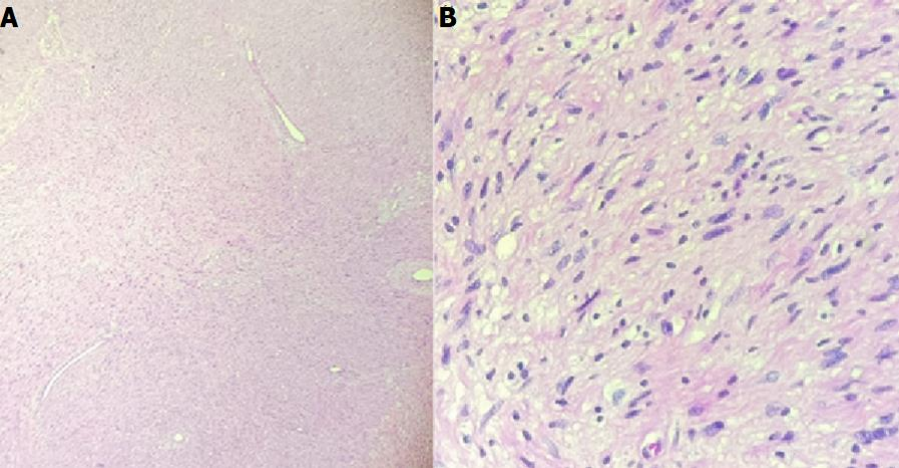
A) microscopic examination showing hypercellular (antoni A) and hypocellular area (Antoni B) (haematoxylin and eosin stain, original magnification x100); B) microscopic examination showing cells are narrow and elongated, nuclear palisading around the fibrillary process (verocay bodies) (haematoxylin and eosin stain, original magnification x400)

Macroscopically, the tumor appeared as a greyish-white mass with a solid-soft consistency. The microscopic examination showed rounded, oval, and spindle cells arranged in a hyperplastic and compact manner, with hypercellular (Antoni A) and hypocellular (Antoni B) areas. Antoni A area was characterized by the presence of Verocay bodies. The tumor cells exhibited eosinophilic cytoplasm, with no evidence of nuclear atypia or mitotic activity. The results followed the characteristics of a benign schwannoma tumor, as depicted in Figure 3.

Based on the pathology examination, the patient was diagnosed with a giant lumbar schwannoma (WHO Grade 1). Furthermore, the Sun and Pamir Classification were utilized to stage the surgical procedure, determining that it was a Group C Type IV tumor, as it was extradural and exceeded 4 cm^3^ in size.

The patient was monitored through outpatient visits. One-week post-surgery, the pain scale measured by the VAS was reported as 1. A Lumbar MRI was scheduled three months after the surgery, which revealed the absence of any enhancing tumor lesions. Therefore, it was suggested to conduct annual follow-up evaluations to monitor for the recurrence of the tumor.

**Patient perspective:** the patient experienced a favorable clinical progression and reported improvement in their symptoms. No complications were reported following the surgery. Furthermore, the patient expressed a high level of satisfaction with the treatment received.

**Informed consent:** the patient provided written informed consent to participate in the study.

## Discussion

Schwannomas are benign tumors that develop from the nerve sheath [2]. Due to their slow growth and the spaciousness of the retroperitoneal lumbar area, they often go unnoticed in their early stage. Furthermore, the symptoms only arise once the tumor causes compression to the nerve root or nearby structures [2]. Intradural spinal schwannomas are more likely to present myelopathic symptoms, while extradural schwannomas usually cause radicular symptoms [3]. The signs of nerve root schwannomas include pain, paresthesia, and numbness in the affected area [3]. In this case, the patient experienced radicular pain due to compression of the L5 nerve root.

MRI is a crucial tool in diagnosing spinal tumors, as it provides a comprehensive evaluation of the tumor's size, origin, and surrounding anatomy. In this case, it was particularly important due to the size and location of the schwannoma. Preoperative radiological evaluation is beneficial for determining the appropriate surgical approach and ensuring a successful outcome. MRI scans of schwannomas showed central and peripheral areas of high and low intensities on T1-weighted images, while T2-weighted images present the opposite pattern. Additional contributions to the investigation can be gained through MRI of the sciatic nerve and Computed Tomography (CT scans) of the spine [4].

Consultation with other surgical departments was deemed necessary due to the location and size of the tumor. A general surgeon and a urologist were consulted, with expertise in retroperitoneal exploration and surrounding anatomical structures. Additionally, a vascular surgeon was consulted in light of the tumor's proximity to the iliac vessels to address potential vascular injuries.

Schwannomas were completely removed while maintaining neurological function. They are typically not firmly attached to adjacent structures, making them relatively simple to take out. However, in this instance, the process was complicated due to the tumor's size, its extension into the retroperitoneal area, and the proximity of significant blood vessels. Obtaining an unobstructed view of the entire tumor can sometimes be difficult, leading to the possibility of residual tissue or damage to nearby structures that could result in significant blood loss [5].

The retroperitoneal approach is utilized for tumors affecting the lower portion of the lumbar spine, down to the L4 vertebral level [6]. The presence of the iliac vessels may pose a challenge for this method, but proper mobilization of these vessels provides a clear view of the retroperitoneal space for accessing the lumbar vertebral body. The patient is positioned in a lateral decubitus position for improved abdominal retraction. The incision for exposing the L4-L5 level starts between the ribs and pelvis and terminates below the umbilicus at the lateral rectus abdominis muscle [6]. When retracting the psoas muscle, caution should be exercised due to its proximity to vital structures, such as the ureter and the sympathetic trunk of the genitourinary system [6]. The retroperitoneal approach provides access to the retroperitoneal space and the L5 nerve root where the tumor originated [6]. The chosen approach depends on the tumor's location and extent. In other documented cases, two-stage surgery was performed, with the anterior retroperitoneal approach utilized in the first stage to dissect the retroperitoneal component of the tumor and laminectomy in the second stage to address the component within the spinal canal [7]. However, in this case, a single-stage surgery was conducted exclusively using the retroperitoneal approach as the mass did not extend into the spinal canal.

The resection technique used was the piecemeal method which violates the tumor capsule and resects the mass piece by piece [8]. The en bloc technique was not used because it had a large mass and was difficult to dissect completely from its surrounding anatomical structures without first resecting part of the mass. It is usually better for reducing the recurrence rate because it doesn't violate tumor capsules, but has a higher risk of injury to surrounding structures [8].

Vascular injury is an issue that could happen during the resection of retroperitoneal schwannoma [9]. The L5 schwannoma was located near important vascular structures such as the common iliac vein. Tumor dissection with a clear view of the operating field will prevent vascular injury. Furthermore, blood product preparation before the surgery is essential in case of suspected vascular injury or severe bleeding [9].

Spinal instability is an issue that can arise in schwannoma with vertebral body erosion. In other cases, it could be caused by schwannoma with massive vertebral body erosion or involvement of facet joints [10]. In this study, the tumor only eroded a small part of the vertebral body, hence, there was no spinal instability. Most of the cases of spinal schwannoma didn't need fusion or instrumentation after the surgery [8].

Schwannoma recurrence is around 4-6%, but several risk factors such as subtotal or partial removal and the tumor size could increase this chance [8]. Patients who underwent the intralesional resection technique also had a higher chance of recurrence than those that had en-bloc resection [8]. The tumor was completely removed in this patient, but the giant size may increase the chance of recurrence. Furthermore, a postoperative MRI is needed to confirm any residual mass or recurrence. Annual MRI and follow-up of the patient's symptoms are also required to observe the tumor recurrence.

## Conclusion

The retroperitoneal giant lumbar schwannoma is a unique case because of its rarity and complexity of management. The treatment goals are complete surgical resection with preserved neurological function and minimal postoperative complications, and these can be achieved through determining suitable surgical approaches and techniques. Meticulous preoperative laboratory and radiology examination should be conducted. A multidisciplinary team could be needed when the tumor extended to an unusual location. Therefore, available resources should be used to maximize the surgery outcome and minimize complications.
